# Association between out-of-pocket costs and treatment patterns in psoriasis and psoriatic arthritis: A claims-based cohort study

**DOI:** 10.1016/j.jdin.2025.11.020

**Published:** 2025-12-04

**Authors:** Alyssa M. Roberts, Charlotte Jeong, Peichi Chou, Elaine J. Ma, Abigail Katz, Matthew J. Yan, Nicole Johnsen, Yvonne Nong, April W. Armstrong

**Affiliations:** a University of Hawaii at Manoa John A. Burns School of Medicine, Honolulu, Hawaii; b University of Arkansas for Medical Sciences College of Medicine, Little Rock, Arkansas; c University of California, Riverside School of Medicine, Riverside, California; d University of Southern California Keck School of Medicine, Los Angeles, California; e Icahn School of Medicine at Mount Sinai, New York, New York; f David Geffen School of Medicine at the University of California, Los Angeles, California; g Division of Dermatology, Department of Medicine, David Geffen School of Medicine at the University of California, Los Angeles, California

**Keywords:** adherence, discontinuation, MarketScan, Medicare, out-of-pocket costs, proportion of days covered, psoriasis, psoriatic arthritis, psoriatic disease, treatment costs, treatment gaps

*To the Editor:* Psoriatic disease often requires long-term therapy, but many patients face out-of-pocket treatment costs not covered by insurance. Prior studies in other chronic conditions have found that higher out-of-pocket costs may affect rates of treatment adherence, defined as the degree to which the patient takes their medication as prescribed, and treatment discontinuation, defined as the patient’s cessation of a treatment before being advised to by their provider.^1^^,^^2^ This study examined associations between out-of-pocket costs and treatment patterns among patients with psoriatic disease using biologic or oral systemic medications.

Patients with psoriasis and/or psoriatic arthritis were identified using the MarketScan Commercial Claims and Encounters and Medicare Supplemental databases (2018-2022) (Supplementary Table I, available via Mendeley at https://data.mendeley.com/datasets/w5tfxybj9t/1). For each patient, out-of-pocket costs were calculated over a 1-year follow-up period by adding deductible, copay, and coinsurance payments made for a study medication (Supplementary Table II, available via Mendeley at https://data.mendeley.com/datasets/w5tfxybj9t/1). Adherence was measured by the proportion of days covered (PDC), defined as the sum of the days supplied with the study medication divided by the 365 days in the follow-up period (≥0.80 classified adherence).^3^ Discontinuation was measured by treatment gaps, defined as the number of days since the patient exhausted their most recent prescription’s supply (≥90 days classified discontinuation).^3^ Medication switches were treated as discontinuations, as our goal was to assess treatment patterns specific to the initial therapy. Relative risks (RR) were derived using an established method based on predicted probabilities from logistic regression and were adjusted for patient characteristics (Table I).^4^

**Table I:** Cohort characteristics of patients with psoriasis and/or psoriatic arthritis, stratified by out-of-pocket cost paid for a biologic or oral systemic medication over 30 days

	Out-of-pocket cost for 30-day medication supply						
	<$15 (*n* = 5679)	$15-99 (*n* = 7013)	$100-249 (*n* = 1982)	$250-499 (*n* = 1332)	$500-999 (*n* = 1303)	≥$1000 (*n* = 1975)	Total (*n* = 19,284)
	*n* (%)	*n* (%)	*n* (%)	*n* (%)	*n* (%)	*n* (%)	*n* (%)
Diagnosis							
Psoriasis only	1295 (22.8)	2840 (40.5)	810 (40.9)	542 (40.7)	556 (42.7)	880 (44.6)	6923 (35.9)
Psoriatic arthritis only	1450 (25.5)	1178 (16.8)	302 (15.2)	208 (15.6)	195 (15.0)	242 (12.2)	3575 (18.5)
Concurrent psoriasis and psoriatic arthritis	2934 (51.7)	2995 (42.7)	870 (43.9)	582 (43.7)	552 (42.4)	853 (43.2)	8786 (45.6)
Age, y							
Mean (SD)	53.6 (12.3)	50.3 (11.6)	49.1 (10.7)	49.1 (10.6)	49.3 (11.2)	48.2 (11.0)	50.8 (11.7)
Sex							
Male	2437 (42.9)	3345 (47.7)	1028 (51.9)	713 (53.5)	684 (52.5)	983 (49.8)	9190 (47.7)
Female	3242 (57.1)	3668 (52.3)	954 (48.1)	619 (46.5)	619 (47.5)	992 (50.2)	10,094 (52.3)
CCI							
Mean (SD)	0.22 (0.67)	0.16 (0.63)	0.13 (0.55)	0.14 (0.55)	0.11 (0.47)	0.12 (0.54)	0.17 (0.61)
Census region							
Northeast	1002 (17.6)	1159 (16.5)	241 (12.2)	164 (12.3)	171 (13.1)	302 (15.3)	3039 (15.8)
Midwest	1527 (26.9)	1396 (19.9)	391 (19.7)	269 (20.2)	268 (20.6)	360 (18.2)	4211 (21.8)
South	1955 (34.4)	2708 (38.6)	849 (42.8)	618 (46.4)	596 (45.7)	952 (48.2)	7678 (39.8)
West	627 (11.0)	699 (10.0)	363 (18.3)	180 (13.5)	183 (14.0)	202 (10.2)	2254 (11.7)
Not reported	568 (10.0)	1051 (15.0)	138 (6.8)	101 (7.6)	85 (6.5)	159 (8.1)	2102 (10.9)
Insurance payer							
Commercial	4772 (84.0)	6543 (93.3)	1942 (98.0)	1303 (97.8)	1248 (95.8)	1927 (97.6)	17,735 (92.0)
Medicare	907 (16.0)	470 (6.7)	40 (2.0)	29 (2.2)	55 (4.2)	48 (2.4)	1549 (8.0)
Insurance plan							
Comprehensive	460 (8.1)	292 (4.2)	38 (1.9)	41 (3.1)	22 (1.7)	17 (0.9)	870 (4.5)
EPO	41 (0.7)	63 (0.9)	36 (1.8)	8 (0.6)	8 (0.6)	20 (1.0)	176 (0.9)
HMO	914 (16.1)	1431 (20.4)	234 (11.8)	123 (9.2)	86 (6.6)	138 (7.0)	2926 (15.2)
Non-capitated POS	304 (5.4)	423 (6.0)	153 (7.7)	105 (7.9)	71 (5.4)	65 (3.3)	1121 (5.8)
PPO	2887 (50.8)	3638 (51.9)	1062 (53.6)	550 (41.3)	552 (42.4)	925 (46.8)	9614 (49.9)
Capitated or partially capitated POS	15 (0.3)	31 (0.4)	4 (0.2)	4 (0.3)	0 (0.0)	1 (0.1)	55 (0.3)
CDHP	533 (9.4)	770 (11.0)	309 (15.6)	172 (12.9)	230 (17.6)	335 (17.0)	2349 (12.2)
HDHP	439 (7.7)	271 (3.9)	113 (5.7)	313 (23.5)	313 (24.0)	449 (22.7)	1898 (9.8)
Not reported	86 (1.5)	94 (1.3)	33 (1.7)	16 (1.2)	21 (1.6)	25 (1.3)	275 (1.4)
Overall treatment class							
Biologic	1354 (23.8)	5738 (81.8)	1819 (91.8)	1234 (92.6)	1154 (88.6)	1826 (92.5)	13,125 (68.1)
Oral systemic	4325 (76.2)	1275 (18.2)	163 (8.2)	98 (7.4)	149 (11.4)	149 (7.5)	6159 (31.9)

*CCI*, Charlson Comorbidity Index; *CDHP*, Consumer-Driven Health Plan; *EPO*, Exclusive Provider Organization; *HDHP*, High Deductible Health Plan; *HMO*, Health Maintenance Organization; *POS*, Point-of-Service; *PPO*, Preferred Provider Organization; *SD*, standard deviation.

Among 19,284 patients, only 38.6% adhered to treatment and 54.4% discontinued treatment over 1 year (Fig 1). Compared to patients paying <$15 for a 30-day medication supply, those paying $15-99, $100-249, $250-499, and $500-999 were more likely to adhere to treatment (aRR (95% CI) = 1.23 (1.14-1.33), 1.32 (1.19-1.46), 1.77 (1.52-2.07), and 1.55 (1.36-1.77), respectively) and were less likely to discontinue treatment (0.87 (0.84-0.90), 0.79 (0.75-0.83), 0.54 (0.51-0.57), and 0.66 (0.62-0.70), respectively). In contrast, patients paying ≥$1000 were less likely to adhere to treatment (0.80 (0.74-0.86)) (Supplementary Tables III and IV, available via Mendeley at https://data.mendeley.com/datasets/w5tfxybj9t/1).

**Fig 1:**
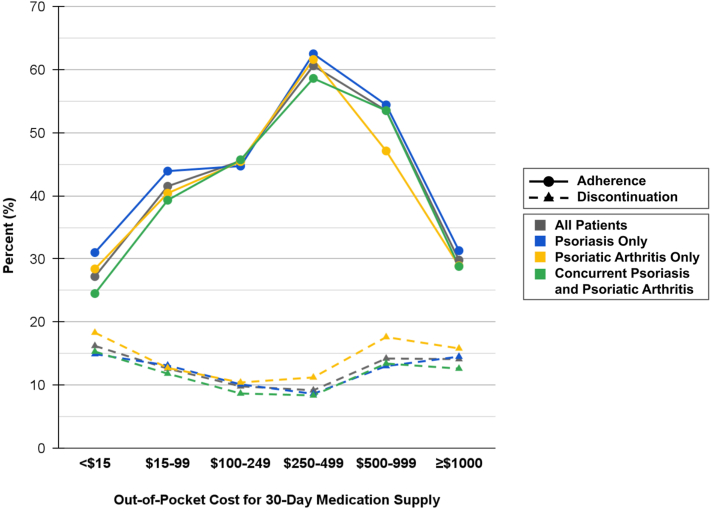
Associations between out-of-pocket costs and adherence and discontinuation rates for biologic and oral systemic treatments among patients with psoriasis and/or psoriatic arthritis.

Importantly, these results should not be interpreted as paying $15-999 improves treatment patterns compared to paying <$15. Rather, our findings may reflect pharmacy benefit manager (PBM) dynamics.^5^ Patients paying $15-999 may be using medications positioned more favorably on formularies due to PBM rebate incentives, enabling greater treatment continuity. In contrast, patients paying <$15 may be using medications outside of PBM-preferred channels, relying on short-term assistance and coverage arrangements more vulnerable to disruption. Additionally, excessive costs may still disrupt care, as patients paying ≥$1000 were significantly less likely to adhere to treatment. Overall adherence and discontinuation rates were also only 38.6% and 54.4%, respectively, indicating that barriers to consistent medication use remain.

In this study, rates of treatment adherence and discontinuation varied significantly by out-of-pocket costs. This research provides valuable insights to guide advocacy efforts aimed at reducing treatment costs and increasing access to care for patients with psoriatic disease.

## Conflicts of interest

Armstrong has served as a research investigator, scientific advisor, or speaker to AbbVie, Alumis, BMS, Galderma, Leo, UCB, J&J, Lilly, Novartis, Sun, Sanofi, Takeda, Regeneron, and Pfizer. Roberts, Jeong, Chou, Ma, Katz, Yan, Johnsen, and Nong have no conflict of interest to disclose.
